# Cancer-associated fibroblasts derived fibronectin extra domain A promotes sorafenib resistance in hepatocellular carcinoma cells by activating SHMT1

**DOI:** 10.1016/j.gendis.2024.101330

**Published:** 2024-05-20

**Authors:** Yan Dong, Yanrong Chen, Yijie Wang, Xiang Zhao, Ruiyang Zi, Jie Hao, Qiong Ding, Haoran Jiang, Xuesong Wang, Fanghao Lu, Houjie Liang, Zhihao Wei, Jianjun Li

**Affiliations:** a Department of Oncology, Southwest Hospital, Third Military Medical University (Army Medical University), Chongqing 400038, China; b Brain Research Center and State Key Laboratory of Trauma, Burns, and Combined Injury, Third Military Medical University (Army Medical University), Chongqing 400038, China

**Keywords:** Cancer-associated fibroblasts, Fibronectin extra domain A, Hepatocellular carcinoma, Serine hydroxymethyltransferase1, Sorafenib resistance

## Abstract

Resistance to sorafenib, an effective first-line treatment for advanced hepatocellular carcinoma (HCC), greatly compromised the prognosis of patients. The extracellular matrix is one of the most abundant components of the tumor microenvironment. Beyond acting as a physical barrier, it remains unclear whether cell interactions and signal transduction mediated by the extracellular matrix contribute to sorafenib resistance. With the analysis of primary HCC organoid RNA-seq data combined with *in vivo* and *in vitro* experiments validation, we discovered that fibronectin extra domain A (FN-EDA) derived from cancer-associated fibroblasts played a critical role in sorafenib resistance. Mechanistically, FN-EDA stimulates the up-regulation of the key one-carbon metabolism enzyme SHMT1 in HCC cells via the TLR4/NF-κB signaling pathway, thereby countering the oxidative stress induced by sorafenib. Moreover, we reinforced the clinical significance of our discoveries by conducting *in vivo* assays with an immunodeficiency subcutaneous xenograft tumor model, which was established using primary cancer-associated fibroblasts derived from clinical HCC tissues, and through the analysis of HCC samples obtained from The Cancer Genome Atlas (TCGA) database. Our findings suggest that targeting the FN-EDA/SHMT1 pathway could be a potential strategy to improve sorafenib responsiveness in HCC patients.

## Introduction

Subtle onset and insufficient diagnostic methods usually result in delayed detection of hepatocellular carcinoma (HCC) in patients until the advanced stage, when surgical interventions are often unsuitable.^1^ Sorafenib is the first-line drug used for advanced HCC patients. Unfortunately, the efficacy of sorafenib is frequently limited by the development of drug resistance in 20%–40% of patients.^2^ Therefore, it is crucial to investigate the mechanisms of sorafenib resistance and develop new combination therapies to improve the prognosis of advanced HCC patients.

Previous studies have demonstrated that cancer-associated fibroblasts (CAFs) contribute to drug resistance by generating the extracellular matrix (ECM) that acts as a physical barrier to tumor cells.^3^ However, it is equally important to explore the potential impact of ECM on the interaction between CAFs and tumor cells, as this interaction may result in molecular-level changes affecting the drug-resistance capacity of tumor cells. Fibronectin (FN), a highly abundant ECM protein, provides structural support and protection to cells while participating in signal transduction processes.^4^ Alternative splicing of three exons from the same gene locus gives rise to various FN isoforms. Among these isoforms, fibronectin extra domain A (FN-EDA) is of particular importance, as it is associated with the development of various tumors.^5^ Our previous studies have revealed that FN-EDA promotes tumorigenesis by stimulating angiogenesis, lymphangiogenesis, and metastasis.^6^^,^^7^ Toll-like receptor 4 (TLR4) is an endogenous receptor for FN-EDA and has been implicated in regulating tumor growth through its influence on cancer metabolism.^8^ However, whether the FN-EDA/TLR4 pathway participates in sorafenib resistance of HCC cells remains unknown.

Apoptosis resulting from oxidative stress is a major antitumor mechanism of molecular targeted therapy. One-carbon (1C) metabolism provides essential precursors for cancer cell growth and maintains tumor cell homeostasis by regulating tumor cells' antioxidant and methylation capacities.^9^ Serine hydroxymethyl transferases (SHMTs), including SHMT1, the cytoplasmic isozyme, and SHMT2, the mitochondrial isozyme, are key enzymes in the 1C metabolism of tumor cells.^10^ SHMT1 influences NADH/NADPH production and DNA methylation status by supplying 1C units for nucleotide synthesis. Additionally, SHMT1 is involved in tumor growth, metastasis, and anti-apoptotic processes in various human cancers.^11^, ^12^, ^13^ However, the specific functions and regulatory mechanisms of SHMT1 in sorafenib resistance in HCC remain unclear.

In this study, we report that the interaction between FN-EDA and TLR4 in the context of CAF-HCC cell interactions promotes the up-regulation of SHMT1 expression in sorafenib-treated HCC cells. This process enhances the antioxidant capacity of tumor cells and contributes to the development of sorafenib resistance. We believe that blocking FN-EDA-mediated SHMT1 activation represents a promising therapeutic strategy that could enhance the anti-tumor efficacy of sorafenib in the treatment of advanced HCC.

## Material and methods

### Human tissue samples

Tissue chips consisting of human HCC tumor and adjustment samples were purchased from Shanghai Outdo Biotech Co., Ltd. (HLiv-HCC150PG-01) and used specifically for analyzing fibronectin.

### Cell lines

The human hepatocellular carcinoma cell lines HepG2 (RRID: CVCL_0027) and Huh7 (RRID: CVCL_0336) were obtained from CHI (Jiangyin) Scientific Co., Ltd. Cells were grown in Dulbecco's modified Eagle medium (Gibco, 11965092) supplemented with 10% fetal bovine serum and antibiotics and maintained in 5% CO_2_ at 37 °C. The medium was replaced three times weekly, and the cells were passaged using 0.25% trypsin/ethylenediaminetetraacetic acid (Hyclone, SH30042.01) and preserved at early passages. All human cell lines have recently been authenticated using STR profiling. All experiments were performed using mycoplasma-free cells.

### *In vivo* tumor models

Eight-week-old NOD/ShiLtJGpt-Prkdc^em26Cd52^Il2rg^em26Cd22^/Gpt (NCG) mice were purchased from the GemPharmatech Co., Ltd. and were acclimated for 2 days. Tumor sizes were measured every three days in two dimensions with calipers and calculated using formula (L × W^2^)/2, where L is the length and W is the width. Seven days after initial cell transplantation, each mouse was orally administered with sorafenib (MCE, HY10201) at a dose of 30 mg/kg every day. Two weeks after the first administration, all mice were sacrificed and tumor tissues were collected. All of our animal studies were approved by the Institutional Animal Care and Use Committee of the Third Military Medical University.

### RNA sequencing data analysis

The single-cell RNA sequencing (RNA-seq) data were downloaded from the GEO database (GSE151530), normalized with Seurat (v4.0.6) in R (R Core Team, Vienna, Austria), and selected as the criteria of the original article (PMID: 34216724). The Harmony package (v0.1.0) in R was used for batch correction. Principal component analysis was performed using differentially expressed genes, and resolutions of 0.8 were explored to optimize subcluster representation. Primary HCC organoids retain the histopathological features of the originating tumor, which makes it an ideal *in vitro* model to clarify the mechanisms that drive sorafenib resistance. The RNA-seq data of paired sorafenib-resistant and -sensitive tumor-derived organoids from four HCC patients were obtained from the GEO database (GSE182593). We performed a transformation of gene expression FPKM values using the following formula: log_2_(FPKM+1). Then, we conducted a normalization process to scale the transformed gene expression values from the same patient to a range of 0–1. This normalization allowed for intra-group integration and inter-group comparisons of sorafenib sensitivity and resistance data from different patients. Gene Set Enrichment Analysis (GSEA) of the RNA-seq gene expression matrix was performed with cluster profile (v4.2.1) in R.

### The overexpression and inhibition of FN-EDA

The overexpression plasmid of FN-EDA was synthesized by Shanghai Sangon Biotech Co. Ltd., Shanghai, China. The gene sequence is AAAGGACTGGCATTCACTGATGTGGATGTCGATTCCATCAAAATTGCTTGGGAAAGCCCACAGGGGCAAGTTTCCAGGTACAGGGTGACCTACTCGAGCCCTGAGGATGGAATCCATGAGCTATTCCCTGCACCTGATGGTGAAGAAGACACTGCAGAGCTGCAAGGCCTCAGACCGGGTTCTGAGTACACAGTCAGTGTGGTTGCCTTGCACGATGATATGGAGAGCCAGCCCCTGATTGGAACCCAGTCCACAGCTATTCCT. As for applying antibody blockade, previously reported monoclonal antibodies against FN-EDA (Sigma, #F6140, RRID: AB_476981) were diluted at a ratio of 1:200 and added to the cell culture solution.^14^

### Western blotting

Cells were lysed using RIPA buffer (Beyotime, P0013) supplemented with a protease and phosphatase inhibitor cocktail (Sigma, P8340, and P2850). Cell lysates were centrifuged at 4 °C for 10 min, and supernatants were used for western blotting. Western blotting was performed using a standard protocol. The antibodies included SHMT1 (Abcam, #ab186130), FN-EDA (Santa Cruz, #sc59826, RRID: AB_783389), and phosphorylated p65 (P-p65; CST, #3031S, RRID: AB_330559), fibronectin extra-domain B (FN-EDB; CST, #36349), H2A (NOVUS, #NB100-56346, RRID: AB_838346).

### Immunofluorescence staining of the paraffin tissue sections

The paraffin tissue section was dewaxed and incubated with 3% H_2_O_2_ at room temperature for 20 min. Then, antigen retrieval was performed using a boiled ethylenediaminetetraacetic acid buffer. After cooling, the slide was blocked with goat serum at room temperature for 60 min and incubated with primary antibodies against SHMT1 (1:200) and FN-EDA (1:200) at 4 °C overnight. Then, the corresponding secondary antibody was added to the slide, which was incubated at 37 °C for 2 h. Nuclei were counter-stained with DAPI.

### Immunofluorescence staining of cells

The cells on slides were fixed with 4% paraformaldehyde for 10 min and permeabilized in phosphate buffer saline for 20 min. Then, cells were blocked with goat serum at room temperature for 60 min and incubated with primary antibodies against FAP (fibroblast activation protein; R&D system, #FAB3715A, RRID: AB_2884010) (1:200) and α-SMA (alpha-smooth muscle actin; R&D system, #MAB1420, RRID: AB_262054) (1:200) for 2 h. Then, the cells on slides were reheated and incubated with the corresponding secondary antibody at 37 °C in the dark for 2 h. Nuclei were counter-stained with DAPI.

### Apoptosis, intracellular ROS production, and NADPH quantification assay

An annexin V-FITC/PI assay (Bestbio, BB-4101) was used to measure apoptotic cells by flow cytometry according to the manufacturer's instructions. After completion of the above treatments, cells were collected by trypsinization and resuspended in 500 μL of binding buffer containing 5 μL of annexin V and 5 μL of propidium iodide at room temperature in the dark for 30 min. After incubation, at least 1 × 10^5^ cells were analyzed on a BD LSRFortessa flow cytometer. Analysis of cytometric data was performed using Flowjo software.

The intracellular reactive oxygen species (ROS) assay was performed using a ROS kit (Beyotime, Shanghai, China). All groups of HepG2 cell pellets were treated as indicated and incubated with fresh medium containing 10 μM dichlorodihydrofluorescein diacetate at 37 °C for 20 min after dissociation and centrifugation. The cells were then washed with phosphate buffer saline three times and then measured within 1 h using ACEA NovoCyte 2050R or BD LSRFortessa flow cytometer. Analysis of cytometric data was performed using Flowjo software.

According to the manufacturer's instructions, NADPH quantification was assayed using a NADP^+^/NADPH assay kit with WST-8 (Beyotime, S0179). Cells were cultured in Dulbecco's modified Eagle medium in six-well plates at 1 × 10^6^ cells per well. Then, 200 μL NADP^+^/NADPH extraction solution and the extracts were centrifuged at 12,000 *g* at 4 °C for 10 min to collect the supernatant. The supernatant (50 μL) was added to the 96 well plates together with working buffer and color development solution and then incubated at 37 °C in the dark for 10 min. Then the absorbance was measured at 450 nm spectrophotometrically.

### Chromatin immunoprecipitation (ChIP)

HepG2 cells were cross-linked with the SimpleChIP Enzymatic Chromatin IP Kit (Magnetic Beads). 540 μL of 37% formaldehyde was added to a 15 cm dish containing 20 mL medium to crosslink proteins to DNA. Then, 2 mL of 10× glycine was added to each 15 cm dish, followed by incubation at room temperature for 5 min. After removal of the media and washing of the cells twice with ice-cold phosphate buffer saline, 2 mL ice-cold phosphate buffer saline with a protease inhibitor cocktail was added to collect the cells. After centrifugation and removal of the supernatant, the cells were immediately subjected to nuclei preparation and chromatin digestion. 1 M dithiothreitol was used to lyse the cells and extract the nuclei and then the lysate was sonicated with several pulses to break the nuclear membrane. Chromatin fragments were immunoprecipitated with anti-P-p65 (Cell Signaling Technology, #71254S) or rabbit IgG (Cell Signaling Technology, #2729S) as control using ChIP Grade Protein G Magnetic Beads (Cell Signaling Technology, #9006). The IP samples were subjected to incubation at 4 °C overnight with rotation for 4 h. The pellet protein G magnetic beads were washed via incubation at 4 °C for 5 min with rotation with a wash buffer. The ChIP Elution Buffer was used to separate the magnetic beads with DNA. After DNA purification according to the centrifugation column method, the immunoprecipitated DNA was quantified by real-time PCR using SHMT1 promoter primers (Table S1). The fold enrichment was calculated by normalizing samples of anti-P-p65 to normal rabbit IgG controls.

### Immunohistochemistry of tissues

Immunohistochemical staining was performed using a Streptavidin/Peroxidase Kit (ZSGB-BIO, SP-9000/9001/9002) following the manufacturer's protocol. Stained tissues were imaged on a Zeiss Axio Imager using Zeiss AxioVision 4.8 software. The immunohistochemical staining results were analyzed using ImageJ software. Initially, the color images were converted to grayscale images, with adjustments made to the contrast. Subsequently, the integrated optical density values for the detection markers corresponding to each sample were measured (FN-EDA in Figure 2E; Ki67 in Figure 5I; FN-EDA and FN-EDB (Invitrogen, # MA5-48023) in Fig. S2B). Finally, statistical analyses were conducted based on the different experimental groups.

**Figure 1:**
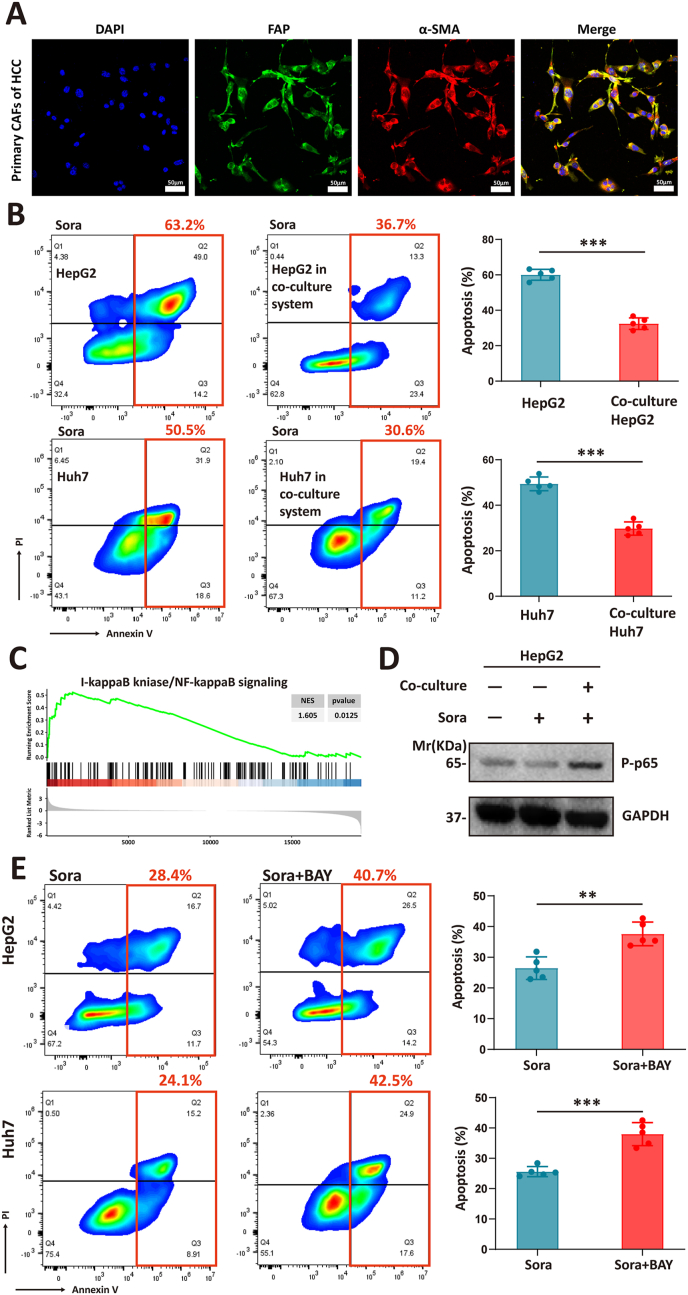
CAFs promote sorafenib resistance by activating NF-κB in HCC cells. **(A)** Immunofluorescence analysis was performed to assess the expression of FAP and α-SMA on primary CAFs. Scale bars, 50 μm. **(B)** Co-culture with CAFs significantly reduced apoptosis of HepG2 and Huh7 upon sorafenib treatment. (blue bar: sorafenib-treated tumor cells cultured alone; red bar: sorafenib-treated tumor cells co-cultured with CAFs). Student's *t*-test of variance, ^∗^*P* < 0.05, ^∗∗^*P* < 0.01, ^∗∗∗^*P* < 0.001. **(C)** Enrichment score of the “I-κB kinase/NF-κB signaling” in sorafenib-resistant group versus sorafenib-sensitive group after sorafenib treatment, analyzed by Gene Set Enrichment Analysis based on the RNA-seq data (after performing log_2_ transformation, normalization, and mean value calculation) obtained from the GEO database (GSE182593). **(D)** Western blot analyses of the protein level of P-p65 in the indicated HCC cells with different treatments. Results are representative of three experiments. **(E)** Inhibition of the NF-κB signaling pathway in HCC cells induced apoptosis significantly upon sorafenib treatment (blue bar: sorafenib-treated group; red bar: sorafenib and BAY11-7082-treated (100 μM) group). Student's *t*-test of variance, ^∗^*P* < 0.05, ^∗∗^*P* < 0.01, ^∗∗∗^*P* < 0.001. CAFs, cancer-associated fibroblasts; NF-κB, nuclear factor kappa B; HCC, hepatocellular carcinoma; FAP, fibroblast activation protein; α-SMA, alpha-smooth muscle actin.

**Figure 2:**
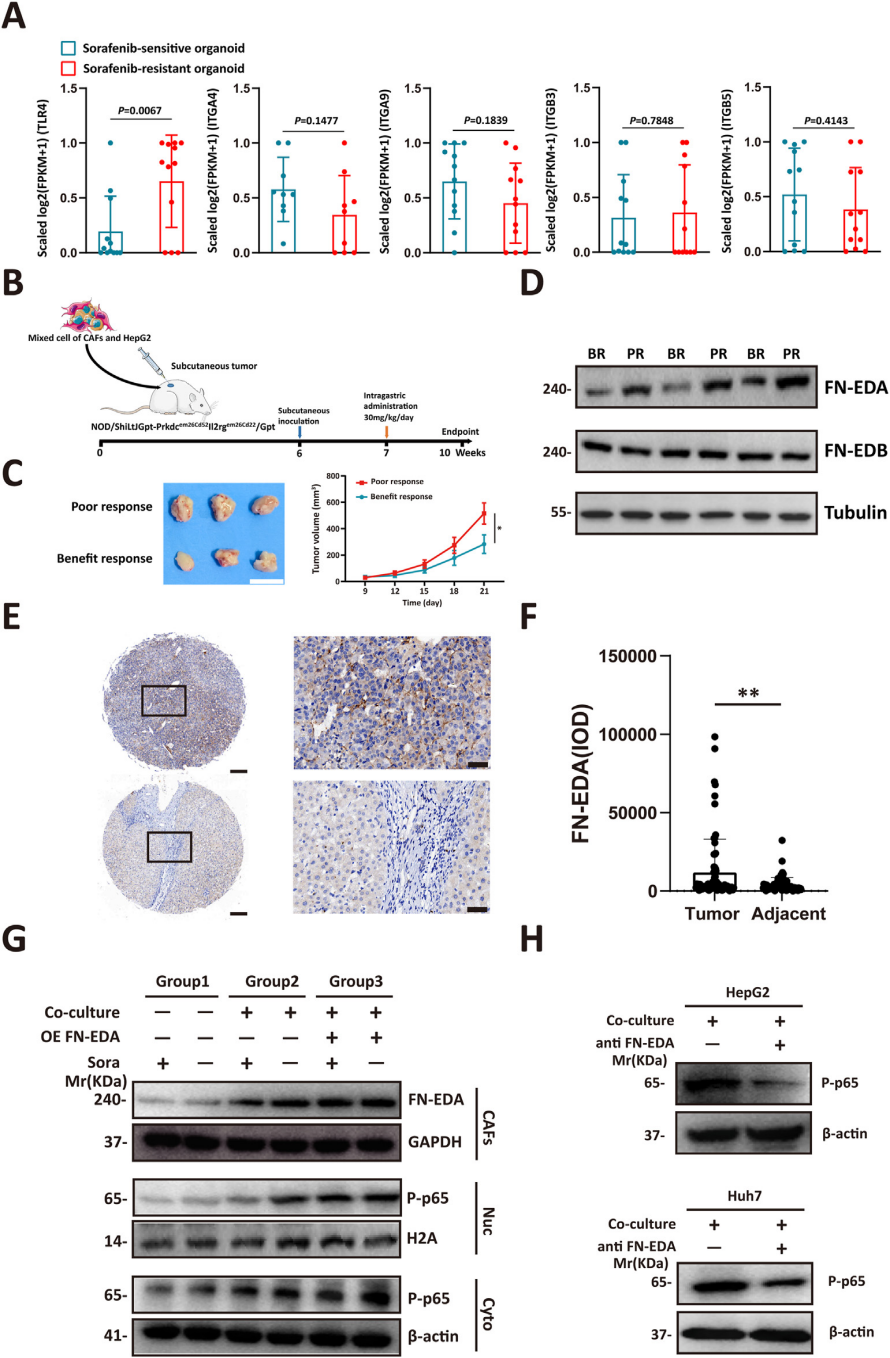
FN-EDA mediates the activation of NF-κB in HCC cells. **(A)** After performing log_2_(FPKM+1) transformation on the RNA-seq data (GSE182593), the gene expression values derived from the same patient were normalized to a range of 0–1. The expression differences of TLR4, ITGA4, ITGA9, ITGB3, and ITGB5 between the sorafenib-sensitive group and the sorafenib-resistant group were then calculated. Student's *t*-test of variance. **(B)** Schematic of the construction of the subcutaneous HCC mouse model. Primary HCC CAFs (5 × 10^5^) and HepG2 (5 × 10^5^) mixed cells were injected into the flanks of mice to form subcutaneous tumors. Each mouse received oral administration of sorafenib treatment (30 mg/kg/day) one week after tumor transplantation. **(C)** Tumor tissues were divided into two groups based on tumor size. The gross appearance of tumors and the tumor volume of each group at the end point were exhibited. Scale bars, 2 cm; *t*-test, ^∗^*P* < 0.05. **(D)** Western blot analyses of the protein level of FN-EDA and FN-EDB in the tumor tissues from (C). **(E)** Representative immunohistochemistry staining for FN-EDA in a tissue microarray containing both HCC tumor and adjacent normal tissues. Scale bars, 200 μm (left panel) and 50 μm. **(F)** Statistical analysis of immunohistochemistry-determined FN-EDA expression level in different samples (*n* = 75). *t*-test, ^∗^*P* < 0.05, ^∗∗^*P* < 0.01, ^∗∗∗^*P* < 0.001). **(G)** We applied the indicated treatment to HepG2 cells and CAFs (the concentration of sorafenib: 10 μM) and measured the expression of nuclear and cytoplasmic P-p65 in tumor cells and FN-EDA in CAFs through western blotting after 48 h. **(H)** Western blot analyses of the protein level of P-p65 in the HCC cells from the co-culture system with indicated treatment. FN-EDA, fibronectin extra domain A; CAFs, cancer-associated fibroblasts; NF-κB, nuclear factor kappa B; HCC, hepatocellular carcinoma; TLR4, Toll-like receptor 4; ITGA4/9, integrin subunit alpha 4/9; ITGB3/5, integrin subunit beta 3/5; FN-EDB, fibronectin extra-domain B.

### Statistical analysis

Data visualization and analysis were performed with GraphPad Prism 9 (GraphPad Software Inc.). The analysis was performed using either Student's *t*-test or one-way ANOVA. Significant difference among groups was assessed as ^∗^*P* < 0.05, ^∗∗^*P* < 0.01, and ^∗∗∗^*P* < 0.001.

## Results

### CAFs promote sorafenib resistance by activating NF-κB in HCC cells

Recent studies have indicated a close association between CAFs and drug resistance in multiple cancer types.^15^ However, whether and how CAFs participate in sorafenib resistance remains elusive. We isolated primary CAFs from surgically resected human HCC tissues (Fig. S1A).^16^ After five cell passages, we confirmed the stable maintenance of the CAF phenotype through immunofluorescence detection, showing high expression of FAP and α-SMA (Fig. 1A). Next, we cultured CAFs in a transwell chamber, while tumor cells were seeded beneath it to form an intercellular interaction system (Fig. S1B). To demonstrate that the interaction between CAFs and tumor cells enhanced the latter's resistance to sorafenib, we assessed the apoptosis levels of HepG2 and Huh7 cells cultured alone and co-cultured with CAFs after 24 h of sorafenib treatment. The results showed a significant decrease in the sensitivity of tumor cells to sorafenib treatment when co-cultured with CAFs (Fig. 1B).

Next, we intended to investigate the mechanism by which CAFs promote sorafenib resistance in HCC cells. The RNA-seq data of sorafenib-resistant and -sensitive organoids from four HCC patients were obtained from the GEO database (GSE182593).^17^ After performing log_2_ transformation and normalization (as described in the *Materials and Methods* section) on the gene expression values from different patients, we calculated the mean expression value for each gene and conducted GSEA. We found that the activation of the NF-κB (nuclear factor kappa B) signaling pathway was a prominent feature of the sorafenib-resistant group compared with the sorafenib-sensitive group (Fig. 1C). Correspondingly, the NF-κB signaling pathway has been proven to play a critical role in the survival and proliferation of tumor cells.^18^ However, it remains unknown whether sorafenib treatment directly activates NF-κB in tumor cells. To address this, we used untreated HepG2 cells as a control and performed Western blot analysis on HepG2 cells treated with sorafenib in both co-culture and monoculture conditions. We found that only HepG2 cells in the co-culture system exhibited up-regulated P-p65 expression, indicating a close association between CAFs and NF-κB activation in sorafenib-treated tumor cells (Fig. 1D). Moreover, we observed that the application of the NF-κB inhibitor (BAY11-7082) significantly increased the apoptotic rate of tumor cells under sorafenib treatment (Fig. 1E). These findings suggest that CAFs promote sorafenib resistance of tumor cells by activating the NF-κB signaling pathway.

### FN-EDA mediates the activation of NF-κB in HCC cells

Next, we aimed to explore the mechanism underlying NF-κB activation in HCC cells. In the tumor microenvironment, CAFs are the primary source of FN, which plays a crucial role in organizing the ECM and facilitating crosstalk between multiple cells.^19^ Among the FN splice variants, FN-EDA and FN-EDB are commonly expressed in the tumor microenvironment and closely associated with tumor occurrence and development.^20^^,^^21^ The main receptors for FN-EDA are α4β1 (ITGA4/integrin subunit alpha 4), α9β1 (ITGA9/integrin subunit alpha 9), and TLR4, while the main receptors for FN-EDB are αvβ3 (ITGB3/integrin subunit beta 3) and αvβ5 (ITGB5/integrin subunit beta 5). By analysis of the RNA-seq data of the aforementioned sorafenib-resistant and -sensitive organoids (GSE182593), we found that only the expression of TLR4 in the sorafenib-resistant group is significantly higher than that in the sensitive group, while the expression of other receptors showed no significant difference between the two groups (Fig. 2A).

To avoid the influence of immune factors on sorafenib resistance, we established a subcutaneous xenograft model by 1:1 injection of HepG2 and CAFs mixed cells in severely immunodeficient NCG mice. Sorafenib treatment was administered to the mice one week after tumor cell inoculation (Fig. 2B). At the endpoint of the experiment, the mice were euthanized, and tumor tissues were extracted. Based on tumor size, the mice were divided into two groups: benefit response and poor response (Fig. 2C). Subsequently, we conducted Western blot and immunohistochemistry analysis on tissues from both groups and observed a significant increase in FN-EDA expression in the poor responsive group, while the expression changes of FN-EDB were not pronounced (Fig. 2D; Fig. S2B). Previous studies have demonstrated a close association between TLR4 and NF-κB activation.^22^ To validate this association, we performed Gene Expression Profiling Interactive Analysis (GEPIA, http://gepia.cancer-pku.cn/) using HCC samples from The Cancer Genome Atlas (TCGA) program database (Fig. S2A). Considering that TLR4 can be activated by FN-EDA rather than FN-EDB,^23^ these results suggested that the FN-EDA generated by CAFs facilitated the activation of NF-κB in tumor cells. Consistently, we performed immunohistochemical analysis of FN-EDA on tissue microarrays and found a significant increase in the expression levels of FN-EDA in HCC tissues compared with adjacent non-cancerous tissues (Fig. 2E, F).

Next, we generated FN-EDA overexpressed plasmid to further investigate the role of FN-EDA in NF-κB activation in HCC cells. By analysis of nuclear and cytoplasmic proteins, we found that co-culturing with CAFs significantly increased the expression levels of P-p65 in tumor cells. Moreover, overexpression of FN-EDA in CAFs further enhanced this effect, suggesting that FN-EDA plays a crucial role in stimulating the activation of the NF-κB signaling pathway in tumor cells (Fig. 2G; Fig. S2C). It should be noted that we also found that sorafenib treatment did not significantly affect the expression of FN-EDA in CAFs, suggesting that FN-EDA could stably activate the NF-κB signaling pathway to promote tumor cell survival under sorafenib treatment (Fig. 2G; Fig. S2C). Furthermore, the addition of a specific monoclonal antibody against FN-EDA to the co-culture system significantly suppressed the activation of NF-κB (Fig. 2H). These findings suggest that CAFs promote the activation of the NF-κB signaling pathway in HCC cells under sorafenib treatment by producing FN-EDA.

### FN-EDA/NF-κB/SHMT1 pathway associated with decreased sensitivity of HCC cells to sorafenib

**Figure 3:**
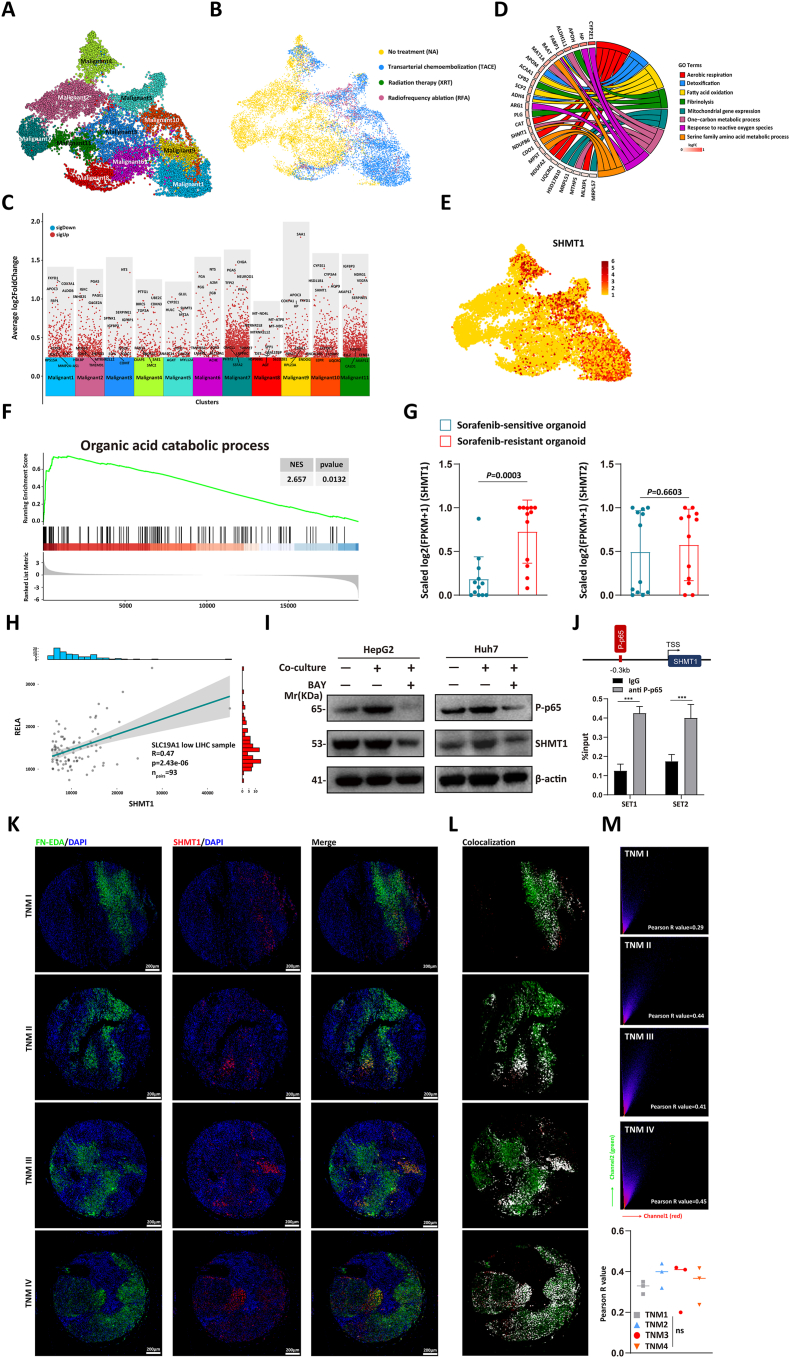
FN-EDA/NF-κB/SHMT1 pathway associated with decreased sensitivity of HCC cells to sorafenib. **(A)** Single-cell RNA-seq data of 25 patients with advanced HCC were obtained from GEO (GSE151530) and integrated with Harmony (v0.1.0). UMAP plot of all 14,288 tumor cells shows 11 clusters. **(B)** UMAP embedding with the integration of transarterial chemoembolization, radiation therapy, radiofrequency ablation, and no local therapy datasets using Harmony (colored by condition). **(C)** The scatter plot shows up-regulated differentially expressed genes from different subclusters in (A); the vertical axis represents the average log_2_ fold change of distinct genes. The *p* value of each gene was <0.05. **(D)** Gene ontology analysis of the top 30 up-regulated overlapping genes in clusters 5 and 10. Two-sided *p* values were calculated using Fisher's exact test. **(E)** The feature plots show the expression of SHMT1 across all subclusters. **(F)** Enrichment score of the “organic acid catabolic process” in the sorafenib-resistant group versus sorafenib-sensitive group after sorafenib treatment, analyzed by Gene Set Enrichment Analysis based on the RNA-seq data (after performing log_2_ transformation, normalization, and mean value calculation) obtained from GEO database (GSE182593). **(G)** After performing log_2_(FPKM+1) transformation on the RNA-seq data (GSE182593), the gene expression values derived from the same patient were normalized to a range of 0–1. The expression differences of SHMT1 and SHMT2 between the sorafenib-sensitive group and the sorafenib-resistant group were then calculated. Student's *t*-test of variance. **(H)** Based on RNA-seq data derived from HCC samples in the TCGA database, we divided all samples into high- and low-expression groups based on the lower quartile expression value of SLC19A1. In the low expression group, we calculated the correlation between the gene expression values of RELA (the coding gene of p65) and SHMT1. **(I)** Western blot analyses of the protein level of P-p65 and SHMT1, respectively, in the HCC cells with indicated treatment. NF-κB inhibitor BAY 11–7082 (100 μM, 48 h). Results are representative of two experiments. **(J)** Chromatin immunoprecipitation-PCR analysis for the P-p65 occupancy on SHMT1 promoters in control HepG2 cells and HepG2 cells treated with BAY 11–7082 (100 μM, 48 h). Data were expressed as mean ± standard deviation. ^∗^*P* < 0.05, ^∗∗^*P* < 0.01, ^∗∗∗^*P* < 0.001. **(K)** Representative immunofluorescence images of HCC tissue samples, with SHMT1 in red, FN-EDA in green, and DAPI in blue. Scale bar, 200 μm. **(L)** Colocalization of FN-EDA and SHMT1 was compared with the Coloc 2 plugin. White represents the areas of colocalization. **(M)** The colocalization correlation coefficient between FN-EDA and SHMT1 was calculated with the Coloc 2 plugin. Statistical analysis of the differences in correlation coefficients between samples from different TNM stages. FN-EDA, fibronectin extra domain A; NF-κB, nuclear factor kappa B; HCC, hepatocellular carcinoma; SHMT1/2, serine hydroxymethyl transferase 1/2; SLC19A1, solute carrier family 19 member 1; ns, non-significant.

Next, we tried to identify the downstream effector molecule of NF-κB. We utilized single-cell RNA-seq data from 25 advanced HCC patients (GSE151530) to investigate the response of tumor cells to cytotoxicity treatments.^24^ The dataset included tumor samples from patients who received local cytotoxic therapy such as transarterial chemoembolization (*n* = 8), radiofrequency ablation (*n* = 6), and radiation therapy (*n* = 2), or remained untreated (*n* = 9). These treatments induce localized apoptosis and necrosis in tumor cells, making them equivalent to targeted therapy. By applying principal component analysis to differentially expressed genes, we identified 11 distinct clusters of tumor cells (Fig. 3A). Notably, we observed a significant difference in transcriptomic profiles between tumor cells derived from treated and untreated patients, with treated cells primarily clustering in groups 1, 4, 5, 9, and 10 (Fig. 3B). Clusters 5 and 10 contained tumor cells from all treatment groups, indicating that these subclusters may possess stronger resistance to treatment.

Then we focused on the up-regulated differentially expressed genes in clusters 5 and 10 and performed gene ontology analysis on the 30 most significant genes shared by these clusters (Fig. 3C). Notably, these genes were enriched in various metabolic pathways, particularly those associated with 1C metabolism and mitochondrial function (Fig. 3D). The 1C unit is primarily generated through the breakdown of serine and glycine. We then examined the expression levels of key enzymes including methylenetetrahydrofolate transferase (MTHFD2), serine hydroxymethyltransferase (SHMT1, SHMT2), and glycine decarboxylase (GLDC) involved in 1C unit generation. Our analysis revealed that SHMT1 showed high expression in tumor cells that underwent local therapy (Fig. 3E; Fig. S3A–C). Traditionally, serine is converted into 1C units through the catalytic activity of SHMT2. However, a previous study has demonstrated that cancer cells with low expression of SLC19A1 (solute carrier family 19 member 1) rely on SHMT1 rather than SHMT2 for 1C unit synthesis.^25^ Consistent with this observation, HCC cells exhibited low expression of SLC19A1 (Fig. S3D). Additionally, our results identified SHMT1 as one of the top 30 differentially expressed genes in clusters 5 and 10 (Fig. 3C). Meanwhile, we conducted CellphoneDB analysis of the interaction between tumor cells and CAFs revealing that clusters 5 and 10 are subgroups of tumor cells that exhibited the most ligand–receptor interactions with CAFs (Fig. S3E). Further screening for ligand–receptor interactions, we found that FN was one of the specific mediators involved in the crosstalk between CAFs and cluster 5/cluster 10 (Fig. S3F). This observation further suggested that CAFs might play a crucial role in treatment resistance by producing FN.

Next, we investigated whether sorafenib treatment led to an increase in the expression of SHMT1 in tumor cells. By analysis of previously mentioned RNA-seq data of HCC organoid samples (GSE182593), we found that compared with the sorafenib-sensitive group, the expression of genes involved in the “organic acid catabolic process” was up-regulated in the sorafenib-resistant group (Fig. 3F). The expression of SHMT1 in the sorafenib-resistant group was significantly higher than that in the sensitive group, while the expression of SHMT2 showed no significant difference between the two groups (Fig. 3G). This further confirmed that SHMT1, rather than SHMT2, played a key role in sorafenib resistance of HCC.

Then, we asked whether the activation of the NF-κB signaling pathway led to the up-regulation of SHMT1. To validate this hypothesis, we analyzed RNA-seq data of HCC samples from the TCGA database and found a positive correlation between the expression of the RELA (the coding gene of p65) and SHMT1 in samples with low expression of SLC19A1 (Fig. 3H). Moreover, we applied BAY11-7082 to the co-culture system and performed Western blot analysis. The results showed that inhibiting NF-κB activation significantly suppressed the up-regulation of SHMT1 expression in tumor cells (Fig. 3I). Next, we examined whether P-p65 could directly activate SHMT1 transcription to promote its expression. Firstly, we performed a bioinformatic analysis of the putative P-p65-binding sites in the SHMT1 promoter and found two putative P-p65-binding sites in a region encompassing 0.3 kb upstream of the transcription start site. ChIP showed that P-p65 bound to the SHMT1 promoter in that region (Fig. 3J). These data indicated that the activation of the NF-κB signaling pathway could directly regulate the expression levels of SHMT1. To further demonstrate the close correlation between FN-EDA and the expression of SHMT1 in HCC cells, we performed immunofluorescence staining on 12 clinical HCC samples of different TNM stages (stages I–IV, with 3 samples per stage) obtained from surgical resection (Fig. 3K). Using the Coloc 2 plugin in ImageJ to analyze the colocalization correlation coefficient, we found a clear colocalization between FN-EDA and SHMT1 (Fig. 3L). The correlation coefficients were around 0.2–0.5, with no significant differences between the various stages (Fig. 3M). The reason that the colocalization correlation coefficient did not exceed 0.5 may be due to the extracellular expression of FN-EDA, which is more diffuse, leading to the colocalization of FN-EDA with SHMT1 not being exactly one-to-one. Collectively, the above results indicate that FN-EDA stimulates the expression of SHMT1 and FN-EDA/NF-κB/SHMT1pathway is closely associated with the sorafenib-resistant capacity of HCC cells.

### SHMT1 promotes the anti-oxidative stress capability of HCC cells

**Figure 4:**
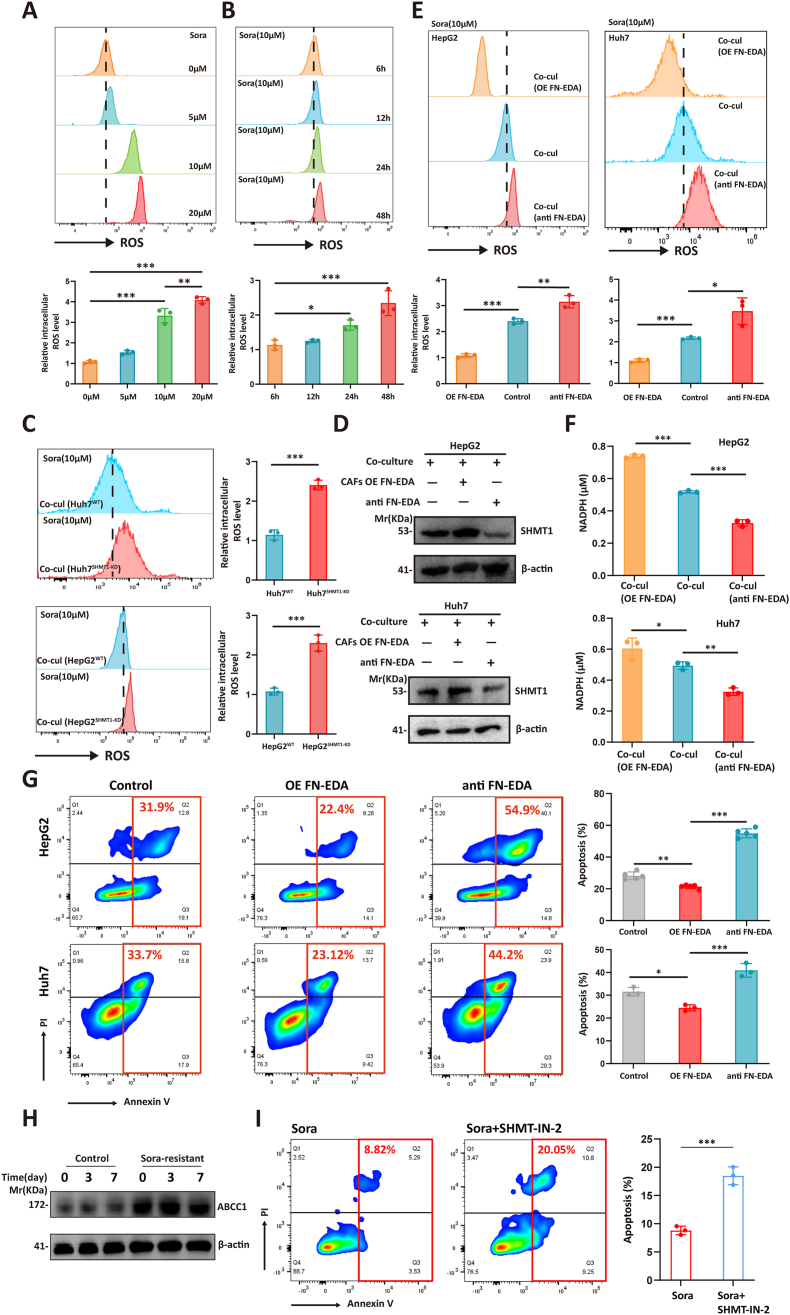
SHMT1 promotes the anti-oxidative stress capability of HCC cells. **(A)** In the co-culture system, different concentrations of sorafenib were applied, and the levels of ROS in tumor cells were assessed 24 h later. One-way ANOVA test of variance, ^∗^*P* < 0.05, ^∗∗^*P* < 0.01, ^∗∗∗^*P* < 0.001. **(B)** In the co-culture system, tumor cells were treated with 10 μM sorafenib, and the levels of ROS in tumor cells were measured at the indicated time points. One-way ANOVA test of variance, ^∗^*P* < 0.05, ^∗∗^*P* < 0.01, ^∗∗∗^*P* < 0.001. **(C)** Wild-type and SHMT1-knockdown HepG2 and Huh7 cells were placed in a co-culture system, and after treatment with 10 μM sorafenib for 24 h, the levels of ROS in tumor cells were measured. Student's *t*-test of variance, ^∗^*P* < 0.05, ^∗∗^*P* < 0.01, ^∗∗∗^*P* < 0.001. **(D)** To determine the expression levels of SHMT1 in HepG2 and Huh7 cells under different co-culture conditions, the following setups were examined: i) Co-culture of HCC cells with wild-type CAFs; ii) Co-culture of HCC cells with CAFs overexpressing FN-EDA; iii) Co-culture of HCC cells with wild-type CAFs, with the addition of monoclonal antibodies against FN-EDA in the co-culture system. **(E)** To assess the levels of ROS in HCC cells under different co-culture conditions (same as the groups in Figure D), the cells were treated with sorafenib, and the ROS levels were measured. One-way ANOVA test of variance, ^∗^*P* < 0.05, ^∗∗^*P* < 0.01, ^∗∗∗^*P* < 0.001. **(F)** The NADPH levels of HCC cells from different groups depicted in Figure D were measured. One-way ANOVA test of variance, ^∗^*P* < 0.05, ^∗∗^*P* < 0.01, ^∗∗∗^*P* < 0.001. **(G)** The apoptosis ratio of HCC cells from different groups depicted in Figure D was measured. One-way ANOVA test of variance, ^∗^*P* < 0.05, ^∗∗^*P* < 0.01, ^∗∗∗^*P* < 0.001. **(H)** The control HepG2 cells and sorafenib-resistant HepG2 cells were both revived from cryopreservation. Once the cells reached a stable condition, cells were collected on day 0, day 3, and day 7 to assess the expression levels of ABCC1. **(I)** The sorafenib-resistant cells were treated with either sorafenib alone (10 μM, 24 h) or in combination with the SHMT–IN–2 (30 μM, 24 h). Subsequently, flow cytometry was employed to analyze the apoptotic state of the cells. Student's *t*-test of variance, ^∗^*P* < 0.05, ^∗∗^*P* < 0.01, ^∗∗∗^*P* < 0.001. SHMT1, serine hydroxymethyl transferase 1; CAFs, cancer-associated fibroblasts; HCC, hepatocellular carcinoma; ROS, reactive oxygen species; FN-EDA, fibronectin extra domain A; ABCC1, adenosine triphosphate binding cassette subfamily C member 1.

During treatment with cytotoxic drugs like sorafenib, cells accumulate ROS, leading to oxidative stress reactions that cause damage to biomolecules such as cell membranes, proteins, and DNA. To ascertain whether oxidative stress-induced damage constitutes a pivotal mechanism behind the anti-cancer efficacy of sorafenib, we exposed tumor cells to various concentrations of sorafenib and different lengths of time. The results demonstrated a proportional increase in cellular ROS levels with the dose and duration of sorafenib treatment, providing evidence supporting the hypothesis that oxidative stress damage contributes to the anti-tumor effect of sorafenib (Fig. 4A and B). Since serine is involved in the folate cycle to generate the important antioxidant NADPH, we hypothesized that SHMT1 may be associated with the anti-oxidant stress response of HCC cells. To test this, we treated the co-culture system with sorafenib and found that the ROS level was significantly elevated in the SHMT1 knockdown group cells (Fig. 4C).

**Figure 5:**
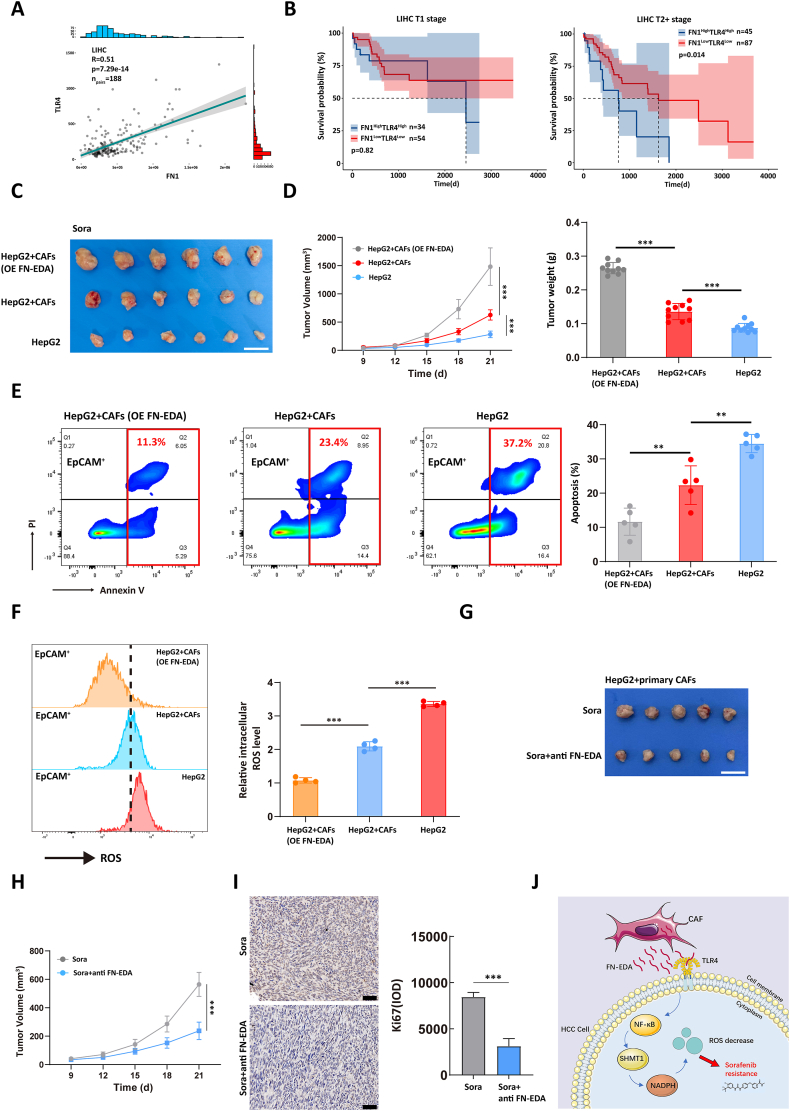
Clinical significance of FN-EDA in patients with HCC. **(A)** TCGA-derived HCC samples were divided into low- (T1, *n* = 115) and high-grade (≥T2, *n* = 188) groups. The correlation between TLR4 and FN1 was calculated in high-grade samples. **(B)** Kaplan–Meier survival analysis of the association between FN1–TLR4 co-expression level and overall survival rate of low- and high-grade HCC patients, respectively. In each stage of the samples, we differentiated high and low expression based on the median values of the FN1 and TLR4 gene expression. Samples with high expression of both genes were categorized as the high expression group (FN1^High^TLR4^High^), while those with low expression of both genes were categorized as the low expression group (FN1^Low^TLR4^Low^). Red line: FN1^Low^TLR4^Low^; blue line: FN1^High^TLR4^High^. **(C, D)** The depicted cells were implanted subcutaneously in NCG mice to establish a subcutaneous xenograft tumor model (*n* = 6/group). One week after cell injection, sorafenib (30 mg/kg) was administered orally to each mouse every day and the tumor size was measured using a vernier caliper. Two weeks after the first administration, all mice were sacrificed, and tumor tissues were collected and weighed. Scale bars, 2 cm. One-way ANOVA test of variance, ^∗^*P* < 0.05, ^∗∗^*P* < 0.01, ^∗∗∗^*P* < 0.001. OE FN-EDA: overexpression of FN-EDA. **(E)** After digesting the tissue collected in Figure C into single cells, we used flow cytometry to detect the apoptosis ratio of tumor cells (EpCAM^+^) in different groups. One-way ANOVA test of variance, ^∗^*P* < 0.05, ^∗∗^*P* < 0.01, ^∗∗∗^*P* < 0.001. **(F)** After digesting the tissue collected in Figure C into single cells, we used flow cytometry to detect the ROS level of tumor cells (EpCAM^+^) in different groups. One-way ANOVA test of variance, ^∗^*P* < 0.05, ^∗∗^*P* < 0.01, ^∗∗∗^*P* < 0.001. **(G, H)** A mixture of 5 × 10^5^ HepG2 and 5 × 10^5^ CAFs was implanted subcutaneously in NCG mice to establish a subcutaneous xenograft tumor model (*n* = 5/group). Mice in each group received the illustrated treatment. Tumor sizes were measured using calipers every three days during the treatment period. Two weeks after the first administration of drugs, all mice were sacrificed and their tumor tissues were collected. One-way ANOVA test of variance, ^∗^*P* < 0.05, ^∗∗^*P* < 0.01, ^∗∗∗^*P* < 0.001. **(I)** Immunohistochemical analysis was performed to assess the expression of Ki67 in each tumor tissue. The left panel is the representative image (scale bar, 50 μm) and the right panel shows the statistical result. One-way ANOVA test of variance, ^∗^*P* < 0.05, ^∗∗^*P* < 0.01, ^∗∗∗^*P* < 0.001. **(J)** Schematic illustration of CAFs-derived FN-EDA promoting sorafenib resistance in HCC cells by enhancing SHMT1 expression. CAFs, cancer-associated fibroblasts; HCC, hepatocellular carcinoma; FN-EDA, fibronectin extra domain A; TLR4, Toll-like receptor 4; FN1, fibronectin 1; SHMT1, serine hydroxymethyl transferase 1; ROS, reactive oxygen species.

Previous data indicated that FN-EDA promoted resistance of HCC cells to sorafenib by up-regulating the expression of SHMT1 in the tumor cells. To confirm the integrity of this pathway, we overexpressed FN-EDA or added a specific monoclonal antibody against FN-EDA in the co-culture system and examined the expression of SHMT1 by western blotting. The results showed a positive correlation between the expression of FN-EDA and SHMT1 (Fig. 4D). Subsequently, we treated the three groups of cells with sorafenib and observed that overexpression of FN-EDA led to an increase in NADPH levels and a lower level of ROS in tumor cells, resulting in the lowest apoptosis rate. Conversely, the group treated with the monoclonal antibody against FN-EDA showed the opposite effect (Fig. 4E, F). To further validate whether FN-EDA enhanced sorafenib resistance in tumor cells, we assessed the apoptosis of tumor cells in the three groups using flow cytometry. The results demonstrated that overexpression of FN-EDA reduced the apoptosis rate of tumor cells, whereas the FN-EDA antibody increased the sensitivity of tumor cells to sorafenib (Fig. 4G). Further, we investigated the impact of interfering with SHMT1 expression on the sensitivity of HCC cells to sorafenib. ABCC1 (adenosine triphosphate binding cassette subfamily C member 1) has been shown to promote sorafenib resistance in HCC cells.^26^ We revived the sorafenib-resistant HepG2 cells previously established in our laboratory and measured the expression of ABCC1 on days 0, 3, and 7 through Western blot analysis. We found that, compared with the control HepG2 cells, the resistant cell line was able to stably overexpress ABCC1, indicating the stability of this cellular trait (Fig. 4H). Subsequently, we treated the resistant cells with either sorafenib alone or in combination with the SHMT1 inhibitor SHMT–IN–2. Flow cytometry analysis of cell apoptosis revealed that the combination therapy significantly increased the sensitivity of the resistant cells to sorafenib (Fig. 4I). These findings indicate that SHMT1 diminishes the susceptibility of tumor cells to sorafenib by bolstering their anti-oxidant stress capacity.

### Clinical significance of FN-EDA in patients with HCC

Considering the ability of FN-EDA to confer resistance to sorafenib treatment in HCC cells, we investigated whether FN-EDA could serve as a prognostic indicator in HCC patients. Since there is a lack of independent coding genes for FN-EDA, we employed the FN coding gene FN1 (fibronectin 1) and the characteristic receptor TLR4 to characterize FN-EDA. To validate this combined characterization, we analyzed HCC samples from the TCGA database and observed a positive correlation between FN1 and TLR4 expression (Fig. 5A). Based on the median mRNA expression levels of FN1 and TLR4, we divided HCC samples from the TCGA database into two groups: FN1^High^TLR4^High^ and FN1^Low^TLR4^Low^. Kaplan–Meier survival analysis revealed that patients with advanced HCC (tumor stage ≥ T2) in the FN1^High^TLR4^High^ group exhibited significantly worse prognoses compared with those in the FN1^Low^TLR4^Low^ group. However, this classification system was not suitable for predicting outcomes in patients with low-grade tumors (tumor stage = T1) (Fig. 5B). These findings suggest that FN-EDA holds promise as a target to improve clinical outcomes in advanced-stage HCC patients.

To further validate the clinical relevance of our findings, we conducted an *in vivo* assay. We divided 18 NCG mice into three groups for subcutaneous tumorigenesis. The first group received an injection of 1 × 10^6^ HepG2 cells, the second group received a mixture of 5 × 10^5^ HepG2 cells and 5 × 10^5^ CAFs, and the third group received a mixture of 5 × 10^5^ HepG2 cells and 5 × 10^5^ CAFs with overexpressed FN-EDA. One week after cell injection, each mouse was orally administered sorafenib at a dose of 30 mg/kg every day, and tumor size was measured using calipers during the treatment period. All mice were sacrificed two weeks after the first administration, and tumor tissues were collected. The results showed that tumors formed by HepG2 cells alone exhibited high sensitivity to sorafenib treatment while co-culturing HepG2 cells with CAFs attenuated the therapeutic effect of sorafenib on tumor cells. Moreover, overexpression of FN-EDA significantly inhibited the therapeutic effect of sorafenib (Fig. 5C, D). Subsequently, we digested the tumor tissues into single cells and sorted the tumor cells using EpCAM antibodies to assess the levels of ROS accumulation and apoptosis rate in each group of cells. The results demonstrated that the tumor cells derived from xenografts containing a mixture of CAFs overexpressing FN-EDA exhibited the lowest levels of ROS accumulation and apoptosis rate (Fig. 5E, F). The aforementioned *in vivo* experiments indicated that FN-EDA could reduce the sensitivity of HCC cells to sorafenib. Subsequently, we continued to utilize *in vivo* assays to evaluate the sensitizing effects of FN-EDA antibodies on sorafenib, thereby providing a potential new strategy for clinical medication. Ten NCG mice were used to establish a subcutaneous xenograft model with a mix of HepG2 and CAFs as previously described. The mice were equally divided into a control group and an experimental group. All mice received oral administration of sorafenib. The experimental group mice were also injected intraperitoneally with FN-EDA antibody (50 μg per mouse) every three days, while the control group mice received an equivalent volume of saline. Tumor volumes were measured every three days to plot the tumor growth curves. All mice were euthanized two weeks after the first administration of the drugs. The results showed that the tumor growth inhibition in the experimental group was significantly better than that in the control group (Fig. 5G, H). All tumor tissues were extracted, and immunohistochemical analysis revealed that the expression of Ki67 in the tumor tissues of the experimental group was significantly lower than that in the control group (Fig. 5I). These results suggested that the combined use of FN-EDA antibody could enhance the sensitivity of HCC cells to sorafenib. These findings highlight FN-EDA as a promising target to enhance the efficacy of sorafenib in HCC patients.

## Discussion

CAFs play a pivotal role in mediating therapeutic resistance in HCC, and elucidating the underlying mechanisms is important for improving patient prognoses.^27^ Modulation of the ECM structure to facilitate the progression of tumors is the major function of CAFs. Recent studies have highlighted the significance of ECM-malignant cell crosstalk in drug resistance.^28^ However, the specific ECM components involved in targeted therapy resistance of HCC remain unclear. In our study, we discovered that tumor cells exploit FN-EDA to induce the expression of SHMT1 via the TLR4/NF-κB pathway. SHMT1 is responsible for serine catabolism, providing the 1C unit required to activate the folate cycle. The subsequent generation of NADPH and glutathione counteracts lethal ROS accumulation, resulting in sorafenib resistance in HCC cells (Fig. 5F).

Due to concealed onset, most HCC patients are diagnosed at an advanced stage where surgical intervention is not feasible. Sorafenib and lenvatinib are FDA-approved first-line drugs for treating advanced HCC. The accumulation of ROS is a key mechanism through which sorafenib and lenvatinib induce malignant cell death. These drugs promote ROS accumulation by inhibiting mitochondrial complex and peroxidase activity and damaging mitochondrial membranes.^29^^,^^30^ However, their clinical efficacy in HCC is modest, with about a one-year extension of median overall survival. Therefore, breakthroughs in drug sensitization are still needed, and attenuating the anti-oxidant stress capability of HCC cells could be an effective strategy. The 1C unit is a well-established driver of the folate cycle and transsulfuration pathway, contributing to the generation of the redox agents NADPH and glutathione.^31^ Hence, 1C unit may affect HCC cells' response to oxidative stress induced by sorafenib treatment, and identifying the initiator of 1C unit production in HCC cells holds significant clinical importance.

Our findings reveal that FN-EDA derived from CAFs is responsible for elevated SHMT1 expression, which is a key enzyme in 1C metabolism, in HCC cells under sorafenib treatment. This result provides valuable insights into drug resistance research: i) The extracellular matrix not only acts as a physical barrier that weakens drug effects by limiting their access to the tumor microenvironment but also serves as a mediator of interactions between various cell types, thereby enhancing the drug resistance of tumor cells at the molecular level. ii) FN-EDA represents an important therapeutic target for HCC. FN-EDA is a soluble signaling molecule that can be detected in plasma.^32^ Future studies should investigate whether circulating levels of FN-EDA can serve as a reliable indicator of the efficacy of targeted therapies for HCC. Additionally, a previous study demonstrated the anti-tumor effect of FN-EDA-targeting chimeric antigen receptor T cells when the tumor stroma expressed FN-EDA, indicating the therapeutic potential of targeting FN-EDA.^33^

In conclusion, FN-EDA emerges as a promising target for both predicting and overcoming resistance to sorafenib treatment in patients with advanced HCC.

## Ethics declaration

The collection of human HCC samples was performed in line with the principles of the Declaration of Helsinki. Informed consent was obtained from all individual participants included in the study. Approval was granted by the Ethics Committee of Southwest Hospital, Third Military Medical University (No. (A) KY202247). All mouse experiments were performed according to the ethical permission of the Animal Care and Use Committees of Third Military Medical University Southwest Hospital, Chongqing, China.

## Author contributions

Jianjun Li, Yan Dong, Zhihao Wei, and Houjie Liang designed the research. Yan Dong, Zhihao Wei, Yijie Wang, Xiang Zhao, Ruiyang Zi, Jie Hao, Qiong Ding, Haoran Jiang, Xuesong Wang, and Fanghao Lu performed the research and analyzed the data. Yan Dong, Zhihao Wei, and Jianjun Li drafted the manuscript. All authors approved the manuscript.

## Conflict of interests

The authors declared no conflict of interests.

## Funding

This work was supported in part by the 10.13039/100014717National Natural Science Foundation of China (No. 81672856, 82203669, 81803028) and the General Program of Chongqing Natural Science Foundation (China) (No. cstc2021jcyj-msxmX0687).

## Data availability

The single-cell RNA-seq data analyzed in this study were sourced from the GEO database (GSE151530). The RNA-seq data analyzed in this study were obtained from the GEO database (GSE182593) and the TCGA database. Other data that support the findings of our study are available from the corresponding author upon reasonable request.
