# Supplementary material for: Cancer-associated fibroblasts derived fibronectin extra domain A promotes sorafenib resistance in hepatocellular carcinoma cells by activating SHMT1
Source: Genes Dis. 2024 May 20;11(6):101330. doi: 10.1016/j.gendis.2024.101330 (PMC11402957; doi:10.1016/j.gendis.2024.101330)
Supplement: Multimedia component 1 [file mmc1.docx]

**
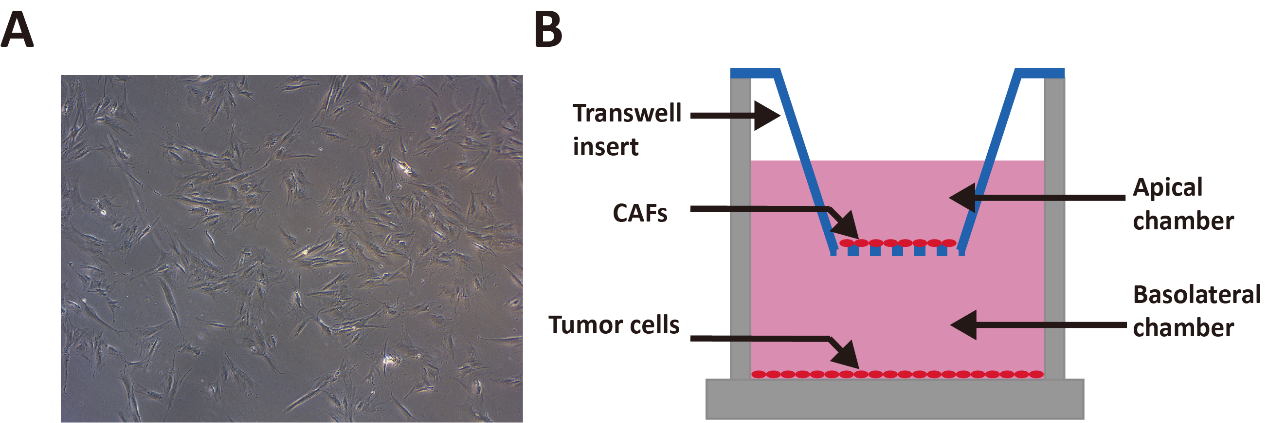
Supplementary Fig 1**

**Supplementary Fig 1. Morphology and intercellular interactions system of CAFs**

(A) The morphological characteristics of primary HCC CAFs.

(B) Schematic of the *in vitro* co-culture system. Placing CAFs in the upper chamber and tumor cells in the lower chamber.

**Supplementary Fig 2
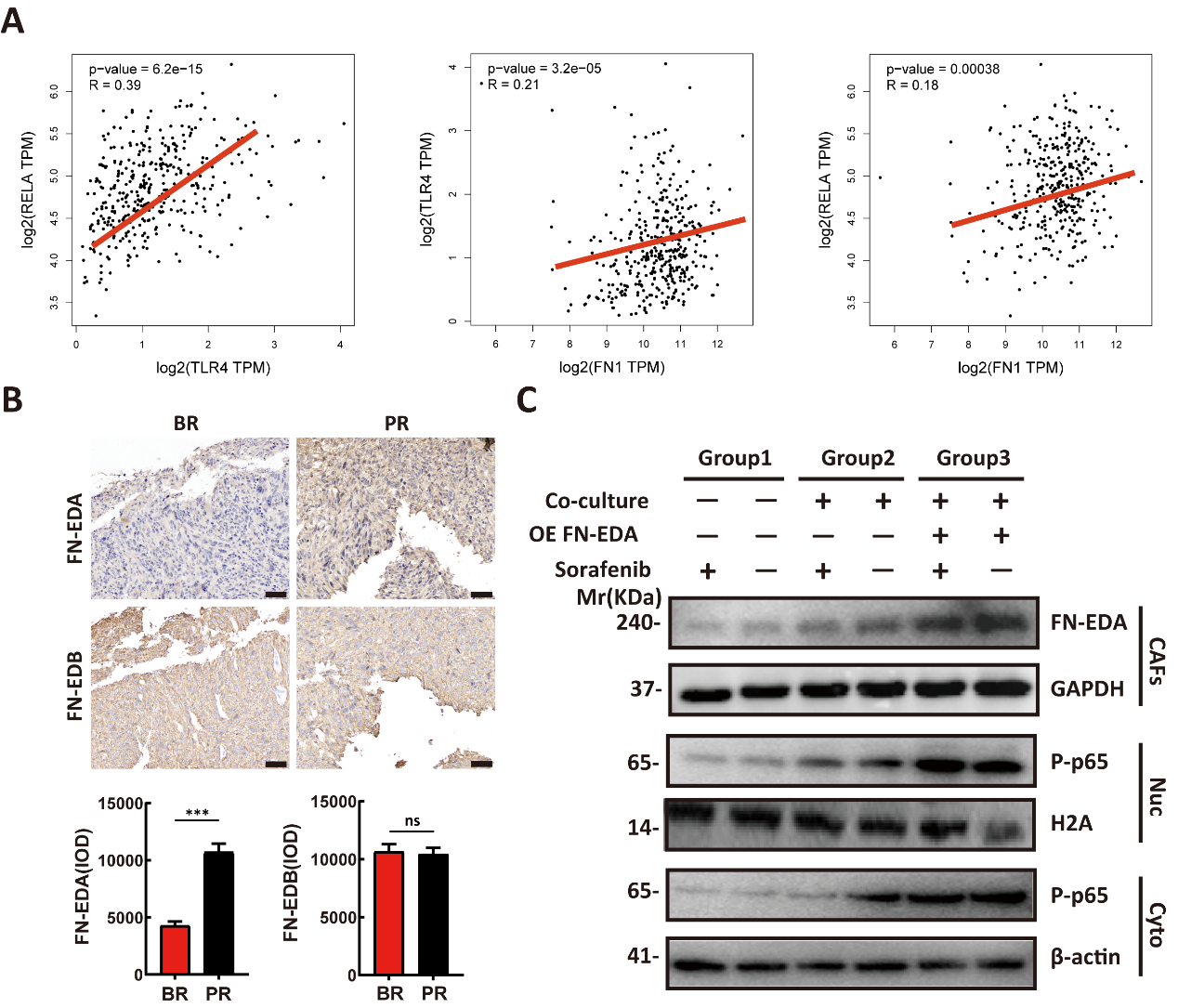
**

**Supplementary Fig 2. FN-EDA Mediates the Activation of NF-κB in HCC cells**

(A) The correlation between indicated genes in patients with HCC was assessed through Spearman analysis (GEPIA, http://gepia.cancer-pku.cn/) utilizing mRNA expression data obtained from the TCGA database.

(B) Immunohistochemical analysis was performed to assess the expression of FN-EDA and FN-EDB in each tumor tissue. The upper panel is the representative image, scale bar=50μm; and the right panel shows the statistical result. Student *t*-test of variance, *P < 0.05, **P < 0.01, ***P < 0.001.

(C) We applied indicated treatment to Huh7 cells and CAFs (the concentration of sorafenib is 10μM), and measured the expression of nuclear and cytoplasmic phospho-NF-κB p65 in tumor cells and FN-EDA in CAFs through western blotting after 48 hours.

**Supplementary Fig 3**

**Supplementary Fig 3. NF-κB/SHMT1 pathway Associated with Decreased Sensitivity of HCC Cells to Sorafenib**


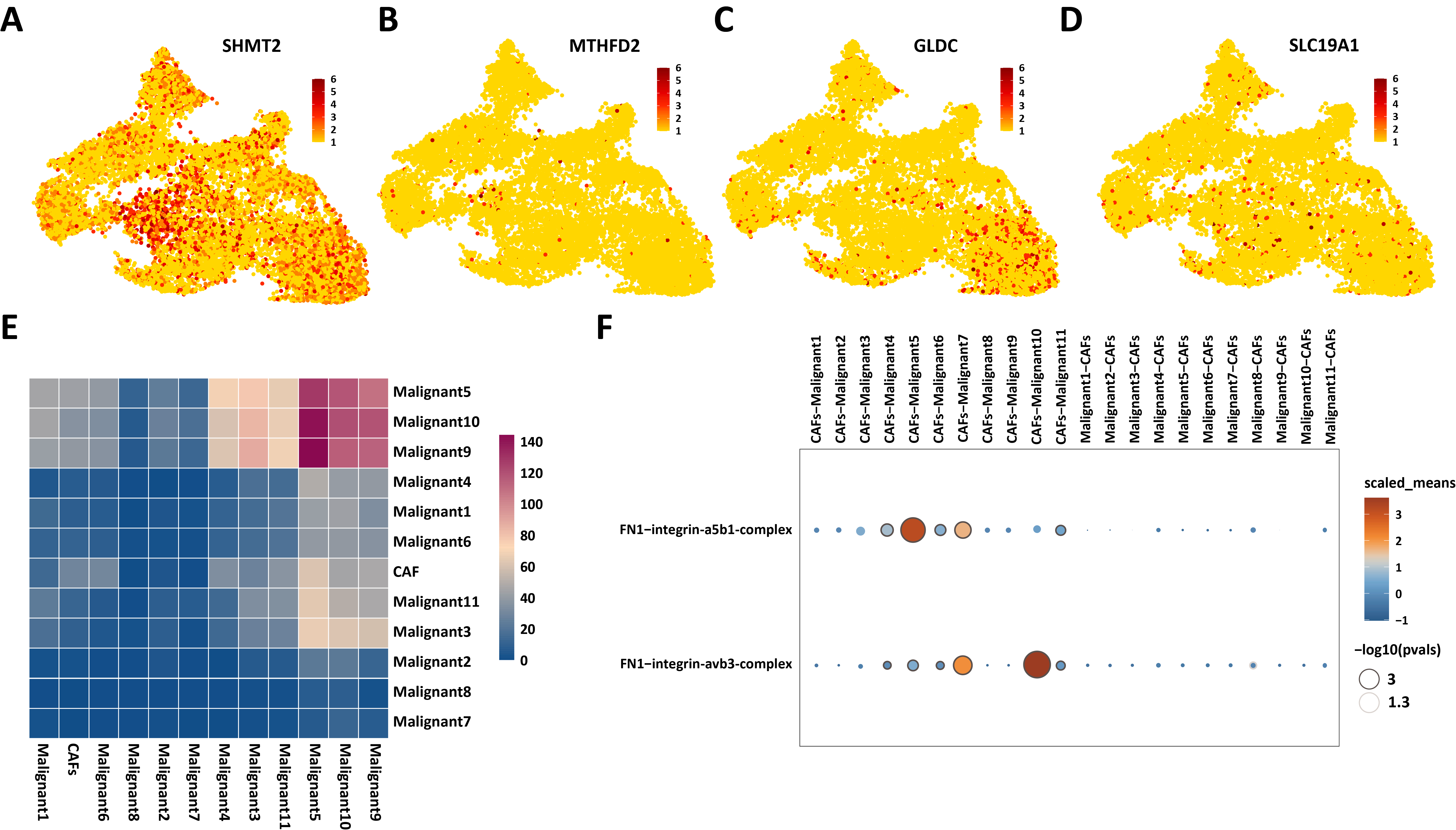


(A-D) Feature plots showing the expression of indicated genes across all subclusters (Related to the analysis of scRNA-seq data in Figure 3).

(E) Representative heatmaps of CellPhoneDB analysis showing the number of ligand-receptor interactions between all kinds of malignant cells and CAFs.

(F) Cell crosstalk based on FN mediated ligand–receptor interaction between malignant cells and CAFs. Red indicates enhanced interaction, while blue represents weakened interaction. Circular outlines are used to indicate P values, with a light gray border indicating a p = 0.05 and a dark gray border indicating a p= 0.001.
